# Relational containment: exploring the effect of family-based treatment for anorexia on familial relationships

**DOI:** 10.1186/s40337-017-0156-0

**Published:** 2017-07-28

**Authors:** Andrew Wallis, Paul Rhodes, Lisa Dawson, Jane Miskovic-Wheatley, Sloane Madden, Stephen Touyz

**Affiliations:** 1 Eating Disorder Service, Department of Adolescent Medicine, The Children’s Hospital at Westmead, Locked Bag 4001, Westmead, NSW 2145 Australia; 2 School of Psychology, The University of Sydney, Sydney, Australia

**Keywords:** Anorexia nervosa, Family based treatment, Family relationships, Attachment

## Abstract

**Background:**

The aim of this research was to investigate the process of familial relationship change for adolescents with anorexia nervosa and their parents, who participated in Family-Based Treatment (FBT).

**Method:**

A Constructionist grounded theory design was employed with purposive sampling. Sixteen young people between 12 and 18 years with a good outcome in FBT and twenty-eight of their parents participated. Young people and their parents took part in separate interviews at the end of treatment. Each interview was transcribed and analysed to identify a unifying phenomenon across the data to elicit a theory that explained the data and then integrated into existing theory.

**Results:**

Prior to treatment families’ experienced significant conflict, disconnection and isolation. The FBT structure, therapist direction, and the specialist medical setting created a process of relational containment. This enabled parents to trust the process of FBT and develop confidence in their executive role in the family. In turn this allowed the adolescent with anorexia nervosa to trust their parents, feel more secure and gradually engage in the treatment process themselves. Improvements in closeness, communication and adolescent sense of self were reported after FBT.

**Conclusions:**

These findings illuminate a possible mechanism of change in FBT. It underscores the importance of parental management of eating disorder symptoms at the commencement of treatment to enable increased parental confidence. Meaningful changes occurred for the adolescents’ that aided normal developmental and relational processes, an important aspect of recovery.

**Trial registration:**

Australian Clinical Trials Register number: ACTRN012607000009415

## Plain English summary

This study used a qualitative methodology to explore adolescent and parental experience of relational change during Family Based Treatment for Anorexia Nervosa. Families often experience disconnection and conflict in the initial stages of the illness impacting on their relationships. We found that Family Based Treatment provided a process that allowed parents to feel confident that they could help their adolescent, and that this led to adolescents feeling more trusting of their parents and more engaged in the treatment process. After treatment there were positive relational changes for adolescents and their family. Better understanding of change processes during treatment can lead to the development of new ideas to improve outcomes.

## Background

Anorexia nervosa is a serious physical and psychological disorder that broadly impacts on both the unwell person and their family [1] and can lead to high levels of psychological distress and conflict for a family [2–4]. The effect of anorexia nervosa on family relationships is becoming increasingly acknowledged [4] with both families and clinicians recognising that anorexia nervosa leads to an accommodation to illness that exaggerates pre-existing family dynamics, impacts current coping, and can lead to feelings of hopelessness and disempowerment [5]. Adolescents with anorexia nervosa, report greater family impairment than their parents at initial assessment [6, 7] with more communication problems being identified by adolescents than their parents [8].

Family processes have also been found to negatively impact progress and outcome. For example, critical comments by parents to their children, while generally low in families with anorexia nervosa, have been related to poor outcome in a number of adolescent treatment studies [9, 10]. In contrast, parental warmth has been related to positive outcome, further demonstrating the importance of relational processes in families where there is anorexia nervosa [11]. A key relational process between an adolescent and parent is attachment security. Anxious attachment anxiety has been shown to negatively impact eating disorder outcome in adults [12]. Prospective studies indicate a link between anxious attachment and eating pathology in adolescents [13] and cross sectional studies indicate reduced attachment security compared to non-clinical controls [14]. So while the impact on outcome for adolescents’ remains unclear, we would expect that anorexia nervosa disrupts attachment processes, as it does family functioning.

When adolescents are impacted by high levels of stress parents play a significant protective role, that helps mitigate the negative consequences, by helping their child feel a sense of security [15]. This role is characterised by adolescents sensing parental availability, emotional attunement and establishing clear behavioural expectations to work within. As with younger children, effective adolescent attachment to parents remains a key correlate of positive psychosocial outcome [16, 17]. These interactions are particularly relevant at times of stress, functioning as a protective mechanism that aids problem solving for the young person [18, 19]. However, given the impact of anorexia nervosa on family functioning, and the often high levels of distress for both adolescents and parents [4], it is unclear whether normal attachment mechanisms, such as help seeking and feeling comforted by parents, or whether parental attunement to the adolescent’s need operates as expected before, during or after treatment.

Family Based Treatment (FBT) [20] is recommended as the first line outpatient treatment for adolescent anorexia nervosa [21]. FBT is supported by a number of randomised control trials [22–27]. The initial focus of FBT is on parents taking control over food and eating, an area of fear and preoccupation for the young person with anorexia nervosa. This may trigger strong negative emotional reactions for some adolescents who may deny their difficulties or see their parents efforts as a threat leading to increasing resistance to treatment [28]. Similarly, the anxiety generated for parents may impact on parental coping, and capacity to respond both firmly and in-tune with their adolescent, reducing family cohesion [5]. These dynamics may prevent the parent-adolescent support mechanism described above from working normally at the commencement of FBT as both parents and adolescents misinterpret each other [13].

Previous qualitative studies investigating family relationships after treatment for anorexia nervosa have indicated a number of changes including increased openness, decreased conflict and better family understanding, although it is also noted that specific family issues and individual needs were not always met [29–31]. However, the majority of these studies have not focused on FBT [20], or the experience of parents and adolescents from the same family together. Additionally, studies have not aimed to understand how these changes occurred.

Understanding relational changes during FBT is important for a number of reasons. Firstly, as noted above, a strong parent-adolescent relationship is protective for both current and future stressors. Post-treatment, both the family and young person will continue to navigate life cycle and developmental issues that require good family support structures to achieve best outcomes. This is particularly critical for adolescents who have had anorexia nervosa as they are likely to have ongoing high psychosocial needs [32]. Therefore, minimising the impact of anorexia nervosa on interpersonal relationships in the family is crucial to future wellbeing. Secondly, learning and understanding more about treatment processes for families who respond to treatment may lead to initiatives to improve the therapy process for families who experience poorer outcomes from FBT or additionally reduce relapse.

The aim of this study was to understand the impact of FBT on family relationships and investigate this process of change for families with a good outcome in an attempt to generate new theoretical insights to guide future enhancements to treatment interventions.

## Method

### Design

Constructionist grounded theory methodology was utilised in this study for its capacity to generate a theory about an area of limited knowledge that also integrates the viewpoint and context of the researchers [33–36]. This was considered important given the researchers pre-existing expertise with the subject matter. This approach differs from the Glaser and Strauss’ [35] original grounded theory method by acknowledging the researchers influence as noted above while still embracing an open ended approach that allows meaning to emerge from the data rather than a pre-existing theory [33]. A strength of this method is the constant comparison of interview data that occurs with each interview until the phenomenon being studied evolves. Purposive sampling was employed to identify participants who had a good response to FBT in order to more fully understand the processes of change that underpin FBT. Purposive sampling is recognised as an effective way to access information rich cases for a particular phenomenon [37]. Good response was defined as completion of the 20-session FBT protocol and weight greater than 85% Estimated Body Weight [38] at session 20.

### Participants

Sixteen adolescents aged between 12 and 18 years and 28 parents who had completed outpatient FBT participated in the study. Fourteen families had two parents, one was a single parent and one had a parent pass away during follow up. All participants met the criteria for DSM-IV Anorexia Nervosa [39].

Prior to commencing FBT, all adolescents had had an inpatient admission and each family in the current study participated in a larger randomised control trial investigating optimal length of hospitalization for 82 medically unstable patients followed by outpatient FBT [25]. Outcomes are reported in detail elsewhere, but briefly full remission after FBT was 31.25% at 12 month follow up, with 87.5% of patients above 85% EBW. Drop out before completion of the treatment protocol was 15.9% [25]. During inpatient care parents were provided weekly psycho-education by the medical team at a clinical update meeting, and adolescents received supportive psychotherapy three times a week for 30 min, focusing on coping with hospital. This ceased at discharge and FBT sessions commenced. Participants completed a 20-session FBT treatment protocol with additional sessions possible if the treatment goals were not met by session 20. Treatment fidelity was confirmed by an author of the FBT treatment manual [25, 40].

All participants were female with a mean age 14.52 years (*SD* = 1.38) at initial assessment. Estimated body weight (EBW) was 80.27% (*SD* = 5.39) with a mean global Eating Disorder Examination (EDE) [41] score of 3.21 (*SD* = 1.15). Parental participation was high with 81% of available parents interviewed. Table 1 describes the participants and clinical outcome at session 20 and at 12-month follow up. Interviews occurred on average 12.11 months (*SD* = 7.80) after FBT ceased.

**Table 1:** Characteristics of interviewees

	Interviewed																	
Subject	Young Person	Mother	Father	Age Admission (yrs)	Age at interview (yrs)	Parental Status	Length illness ((mths)	% EBW at admission	%EBW at start of FBT	%EBW at session 20	%EBW at 12 m follow-up	Global EDE at admission	Global EDE at session 20	Global EDE at 12 m follow-up	Remission at session 20	Remission at 12 m follow-up	P3 (Y/N)	Number of sessions
1	1	1	1	18.05	20.05	Married	18	84.94	92.24	90.24	98.11	1.3	0.34	0.09	0	1	Yes	23
2	1	1	0	13.49	15.54	Married	12	79.46	87.44	100.67	100.49	4.49	0.64	0.12	1	1	Yes	38
3	1	1	1	16.10	18.42	Married	3	85.61	91.09	89.43	89.00	4.53	4.02	0.74	0	0	Yes	63
4	1	1	1	13.68	16.24	Married	6	75.46	78.75	89.31	91.36	1.3	0.34	0.09	0	0	Yes	23
5	1	1	1	13.48	16.25	Married	8	83.37	92.85	87.20	87.15	4.5	0.26	0.65	0	0	No	40
6	1	1	1	14.62	18.18	Married	12	80.27	90.10	94.42	95.77	5	1.41	0.78	0	1	Yes	36
7	1	1	0	14.23	16.94	Married	6	78.70	83.86	94.05	89.11	1.75	1.15	0.73	0	0	Yes	26
8	1	1	1	15.09	17.72	Married	6	79.77	93.29	94.31	112.64	3	1.03	0.43	0	1	Yes	30
9	1	1	1	14.03	15.77	Married	12	83.86	92.32	103.85	88.43	2.86	0.31	0.76	1	0	Yes	38
10	1	1	1	13.04	15.70	Married	5	84.89	95.09	99.99	119.58	3.145	4.39	4.7	0	0	Yes	48
11	1	0	1	12.86	14.47	Widowed	6	85.90	95.72	113.62	119.88	3.63	1.62	1.138	1	1	Yes	25
12	1	1	0	15.29	17.48	Divorced, Single	6	82.52	91.69	96.52	102.23	3.325	2.35	1.813	0	0	Yes	46
13	1	1	1	13.74	15.48	Married	6	79.82	90.28	97.85	104.14	2.94	1.28	0.16	1	1	Yes	26
14	1	1	1	15.61	13.32	Married	5	79.82	90.28	97.85	104.14	2.94	1.28	0.16	1	1	Yes	32
15	1	1	1	15.70	17.43	Married	9	75.78	92.01	100.22	110.37	4.21	0.19	0	1	1	Yes	10
16	1	1	0	13.36	14.28	Married	3	64.20	78.08	89.81	85.94	2.37	0.88	0.81	0	0	Yes	26
		MEAN		14.52	16.45			80.27	89.69	96.21	99.90	3.21	1.34	0.82				33.13
			SD	1.38	1.73			5.39	5.21	6.73	11.31	1.15	1.26	1.14				12.52

Note. Full remission criteria - weight at or above 95% EBW and Global EDE within 1 SD of community norms. EBW, estimated body weight; EDE, eating disorder examination; P3, completed FBT phase 3

All participants no longer met weight criteria for anorexia nervosa at session 20 or 12 month follow-up (>85% EBW). Five participants remained below 95% EBW at 12 month follow-up, but all these patients had a global EDE in the normal range (<1.74, within 1 standard deviation above community mean [42]. One participant had a global EDE score in the clinical range at 12 month follow up but their weight was 102.23% EBW. The mean number of sessions was 33.13 (*SD* = 12.52) but the range broad 10–63 sessions. All but one participant completed all three phases of treatment.

#### Recruitment

Families who completed the 20-session protocol with weight greater than 85% EBW were invited to participate during the RCT follow-up period until data saturation occurred. To be included at least one parent and the young person needed to complete the interview, 6-months or more after completing treatment. The coordinator of the RCT provided information about the research to interested adolescents and their parents meeting inclusion criteria. Of the 17 families approached none declined, however one young person decided not to be interviewed and this family was withdrawn leaving 16 participants. This study was approved by the Human Research Ethics Committee of Sydney Children’s Hospitals Network, Westmead Campus (2006/114). Participants gave informed written consent prior to interviews.

### Procedure

In-depth, face-to-face interviews were conducted with each young person and their parent(s) separately. Interviews lasted between 30 and 60 min and were audio recorded and transcribed verbatim. Each interview began by asking participants about their experience of outpatient FBT treatment and then focused on parent-adolescent relationships before hospitalisation, during FBT and after treatment. This approach was thought to better isolate the impacts of FBT by comparing before and after treatment. During the interview participants were encouraged to describe and explore the meaning of their treatment experiences. After the first two families were interviewed a format developed that asked participants to describe family relationships before anorexia nervosa, prior to treatment, during FBT and after FBT. The questioning process was similar for parents and adolescents with each question in the interview building on the previous response so as to enable a conversation that generated more meaningful and spontaneous responses. Interviews became increasingly focused as they progressed in line with grounded theory methodology [33].

### Data analysis

Each interview was analysed using grounded theory principles [36]. Analysis was progressive using a constant comparative method. Initial codes from the interviews were clustered and a second level of focused coding completed to develop a core category and explanatory framework. Throughout the analysis process, memo writing was used as an analytical strategy to develop high-order concepts and identify links between codes and categories, while remaining true to the data. To ensure rigor throughout the process of analysis the first two transcripts were cross-coded by two of the authors (AW, PR). Participants own words and direct quotes were used to illustrate the model thus grounding the theory in the data [43, 44]. Findings from the analysis were compared with the literature to place the results for this group in a wider theoretical context [45]. An additional credibility check involved consulting two adolescents (18 and 21 years at consultation) and one mother about the results [43]. Consultants were not participants in the study but had similar attributes to participants in the current study (for example, they had recovered from a similar severity of anorexia nervosa, received FBT and no longer met criteria for anorexia nervosa). Consultants confirmed the theoretical construction and a good fit with their own family narrative.

In addition, constructionist grounded theory method acknowledges the researchers’ influence in design and analyses [33, 46, 47]. This study was developed from observing the positive effect of FBT over many years on young people and their families after treatment, as well a detailed understanding of adolescent development processes and attachment relationships. The first author (AW) has more than 20 years experience working with adolescents and more than 10 years experience implementing FBT in a specialist eating disorder service.

## Results

**Fig. 1:**
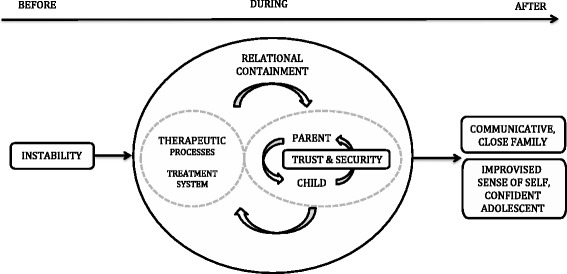
Relationship change process in FBT

Constructionist grounded theory was used to develop a theoretical understanding about relationship changes and FBT. Results are described in three stages as before inpatient treatment, during FBT and after treatment. Participant’s key phrases are in italics, and quotes are used to support theoretical development. The letter and number after each quote denote role (adolescent, mother, father) and participant number. The change process has been termed relational containment and is described below and depicted in Fig. 1.

### Before inpatient treatment

There was a strong acknowledgement from both parent(s) and their adolescent that interpersonal relationships suffered within the context of the illness before treatment and this continued into the early treatment process. Families described instability in this period with themes of conflict, disconnection, isolation and reduced confidence.

#### Conflict and disconnection

Adolescents were clear that relationships with parents had become stained, tense, stressed and that normal conversation was difficult. A number of adolescents described being consumed by thoughts of conflict. Parents emphasised meal conflict as being particularly difficult but also observed conflict between the adolescent and their siblings, as well as conflict between themselves as to how they should help their daughter. Conflict led to relationship disconnection: *“…they (relationships) got really tense because like it was stressful for me and it was like vicious for my parents so I don’t think they really knew so much what to do like before we started coming here” (A5).*

#### Isolation and reduced confidence

Notable for all adolescents was feeling isolated from their family and disconnected from parents: *"I just like I kind of made my own little corner I wouldn’t let anyone in" (A14)* and *“we weren’t being like a family, a bit separated, like I was separated from everyone else in the family” (A10).* Adolescents’ described feeling worthless and angry, with one young person describing it as like being a stranger. A number of adolescents described deliberately wanting to isolate because it was painful to be physically near their parents but feel emotionally distant. There was a general awareness of the impact of isolation, with one adolescent describing this insightfully as *undermining trust and security.* Overall adolescents’ reported being unable to share with or turn to parents for emotional support because of the anorexia nervosa.

Similarly, parents noted changes from their perspective with their adolescent becoming more emotionally needy, experiencing profound distress and observing the emotional impact on siblings. They also noted reduced confidence not only for their adolescent but also in their own approach as parents describing, avoiding conflict, family isolation from outside supports and feeling *devastated and powerless* with a problem that was beyond *instinct and common sense*, with one mother saying *“I just think that we were all just really sad like and just feeling helpless”* (*M16).*

### During FBT

Three themes were identified during treatment as contributing to relationship change; therapeutic processes including FBT concepts, trust and security, and treatment system as backup. Fig. 1 depicts the interaction between these themes as two circles mutually influencing each other achieving a change process as explained below.

#### Therapeutic processes

Both parents and adolescents identified processes in therapy that were significant in building relational containment. The structure of FBT with regular appointments, set treatment sequence, the initial intensity and consistent support created a sense of stability, and expectation of progress. This resulted in less uncertainty, as goals were set and progress checked. For example, *“Treatment gave me tools and framework and a structure and permission I suppose” (M12).* Parents felt more secure with this high level of accountability and structure, describing the therapy like a road map. Both parents and adolescents identified the therapist in the crucial role of mediator, guide or coach. However, more than this parents gained confidence from the therapist’s reassurance and clear directives. In addition, adolescents identified the therapist as giving the parents tools to help such as “*the program gave like a structure for my parents and a structure for me so they knew that they weren’t alone. So I think that was a massive positive thing”* (*A8).* They described the therapist with a neutral quality using questions, being suggestive and having a presence that aided communication such as *"I think it helped us like when we'd come here because I wouldn’t be talking a lot at home so then it would be the time we'd actually like communicate" (A11).*

Parents also identified key FBT concepts during the interviews that increased their confidence. Most frequently identified was the initial focus on parental management of eating disorder symptoms, the importance of parents working together, the role of sibling(s), and externalising anorexia nervosa. For example one mother commented on externalising, *“…it was constantly, ‘well are you going to listen to anorexia or are you going to bring it out as a separate entity’, I think that was really helpful” (M6).*

The above elements provided stability and confidence for parents that allowed them to approach their young person less impacted by the effect of anorexia nervosa. In contrast to before treatment, parents generally described becoming more open and seeing each other’s perspectives because of the discussions in session. This translated to less fractured communication between parents, and between parent(s) and their adolescent, as the FBT sessions clarified plans and encouraged clear communication. This occurred at varying levels for individual families but as long as there was some positive change, this seemed to help treatment progress. Parents described an awareness that they wanted to convey a relationship message using words during the research interview such *unconditional love*, *commitment* and *fighting for her.* One mother reflected on her daughter’s awareness of parental commitment reporting she said to her *“…I couldn’t have done this without you and you’ve saved my life” (M12).*

#### Trust and security

Responding to the parent actions, the adolescents’ seemed to understand the adults’ intention to help. The adolescents expressed this in different ways. Some of the adolescents’ could clearly describe the need to be challenged with eating to recover, and knowing this could not be done in isolation or without emotional difficulty. For example, one adolescent said “…*it slowly got better and we got closer and we worked together and then once we got better it was like we forgot about all the arguments and we were just close again” (A13).* While another described trust replacing control: *“I felt the need to be in control but as soon as they took one of them away from me you know I sort of had to put my trust in to them that they were going to take care of me” (A3).*

Still others identified that seeing their parents hurting or getting to know them in a different way was part of the change process that drew them away from the anorexia nervosa. For example, *“I hated seeing them upset because of me and my acts and stuff like I’d rather just everyone be happy”* (A13). What allowed for these changes seems to have been an increase in trust and security between parents and child, put well by one adolescent *“I understand that if she says I love you she really does mean it because like I use to think that she use to just say that but it’s not like that at all” (A2)*. The combination of being challenged to recover and parent attunement seems to have created a safe secure space for adolescents. One adolescent said *“…they tried to be as understanding as they could be (parents) …they were kind of like ‘if you need us we’re here let’s just talk about it and I think it helped because for the first time I didn’t actually shun them, I went to them…it’s so weird cause for the first time in my life I let myself depend on some one else*” *(A1).*

This increased security was described in a number of ways such as understanding each other again, parents not being critical, learning to provide emotional support, reassurance and feeling that parents were *there for me*. This was also described as parents being *constant* and the family becoming more *capable* and *compatible.* Interestingly, the adolescent’s point of view developed in the context of parents being firm around eating disorder behaviours and the adolescents seeing firmness as love, which helped make it easier to *give up*. This reciprocal process was put thoughtfully by one adolescent noting the power of the attachment relationship to support change; *“I don’t think mum would have been able to do it without N (therapist) but N kind of gave mum the tools to do it…mum just kept doing what N was doing and it seemed to work better when mum did it…N had the right ideas and everything but you just need the right person…I think it has a lot to do with relationships um you can’t run a away from your mum…” (A2).*

#### Treatment system as backup

While not as critical to change as the therapeutic processes noted above, hospital admission and medical appointments were identified as helpful in two senses. The first was as an initial catalyst for change. For example, one mother identified how hospital stopped her daughter’s isolation; *“she just started talking all of a sudden it was like this big release as in someone’s about to help me” (M7).* Secondly, it gave parents the authority to act: *“there was a voice of authority (hospital) saying this is the way to approach it” (M5)*.

### After treatment

After treatment, themes of closeness and communication, and sense of self were identified for most families. It did appear that significant life events for two families differentiated them from others. One father had a significant trauma history and there was no relational change with the young person. In the other family, the mother passed away during treatment and this event was still having an acute effect on the adolescent and father’s relationship. Despite these challenges, both young people noted changes in how they felt about themselves.

#### Closeness and communication

Adolescents consistently identified improvements in closeness and communication with parents: *“I guess I’ve become more confident in um talking to my parents about little problems and things like that” (A4)*. Improved closeness was a consistent disclosure but was targeted for some adolescents on particular relationships, such as a particular parent or sibling. For example, “*um I understand that she (mother) says she loves you she really does mean it because like I use to think that she use to just say that but it’s not like that at all” (A2).* This was described well by one adolescent as no longer feeling a disconnection*.* The level of insight into how changes occurred varied with some adolescents identifying treatment and others recovery. Parents described similar changes but also noted changes in the parenting dyad as stronger or closer, for example, *“It’s taught everyone even (our daughter), even though she doesn’t see it that you know just how strong support can be, and that it is there and always was there” (M9).*

#### Sense of self

Adolescents described a number of personal changes that had occurred during the therapy. The first was an improved sense of self and increased confidence. This ranged from describing self-acceptance, to feeling parental love and being less self-critical. Secondly, adolescent’s identified a new capacity to trust, accept help and depend on another when needed. One adolescent said *“my trust in my parents helped me trust other people and trust myself as well actually” (A3).* Significantly, the change in self seemed to occur through trusting parents or indirectly by the comfort of parental presence. For example *“I guess I like having my parents as sort of like a safety net, I don’t know I really like being able to talk to them about stuff, like I’m really stressed out or whatever it makes me feel a lot better being able to talk to them” (A5).* Parents noted similar changes in their adolescent and described this in terms of confidence, resilience and being more emotionally expressive, for example; *"I think its made her a bit of a stronger person and um more resilient to things" (M7).* Most families noted an overall change in parenting style such as having firmer boundaries, and intrapersonal change described as increased backbone, resilience or reassessment of their own beliefs. For example *“I think going through this journey with (young person) has made me more resilient” (M12).*

## Discussion

The aim of this study was to investigate the impact of FBT on familial relationships for those who had a good treatment response, and develop a theory to explain any changes. The results indicate a number of changes occurred including improved communication, closer family relationships and intrapersonal change for adolescents and parents. Change occurred through a combination of therapeutic processes and the parent-child attachment system. We have termed this phenomenon - *relational containment*. In summary, the relational change process occurred through containment of parental anxiety by the therapeutic process, and wider treatment system, which included the initial hospitalisation. This increased parental confidence to manage anorexia nervosa, but also to relate to their young person in a more attuned way that was emotionally containing. This began, and was aided by the therapy process. As a result, the young people became more open with parents in therapy and at home. Over time this resulted in an improved feeling of security and trust. Adolescents described feeling more understood and in a shared process, working together with their parents. This resulted in the adolescents developing empathy for their parents’ situation, and reduced previously felt isolation. Over time this allowed adolescents to engage in the therapy. Relational containment is a reference to Bion’s theory of ‘container contained’ [48, 49] where the parent/therapist can take intolerable and fearful feelings and return them to the parent/child in a way that is manageable. In simple terms, the treatment process was the container for parents, which increased parental capacity. Consequently, parents then provided the container to their child and thus the young person’s felt security improved.

A key finding was confirmation from both parents and adolescents that the therapy process provided opportunities for improved family communication. The significance of this seemed to lie in how this allowed the meaning of family members behaviour to be clearly understood by each other i.e., parents acting out of love and concern and young people needing support. Ultimately this resulted in improved or restored trust between adolescent and parent(s), which became a mutual process as adolescents then empathised with their parents position and began to experience communication working better. Better communication also appeared to reduce resistance to parental control, as parents became more attuned. Previous studies have reported that adolescents recognise that parental control of food is an important step to recovery [30, 50] but what might reduce resistance has not been previously investigated.

The therapist role was significant for both adolescents and parents in mediating this process. Previous studies have identified the importance of the therapeutic relationship for parents but this has been less clear for adolescents [51–53]. The therapeutic relationship had a more direct effect initially for parents than the adolescents, by increasing their confidence and knowledge. This probably commenced in the hospital environment, was aided by the therapists being part of the multidisciplinary team, and then strengthened in outpatient care. This is important as an increased parental self-efficacy has a positive effect on treatment outcome [54, 55]. However, the adolescents relied more on the therapist mediating the communication with parents rather than on any direct support the therapist gave. This may be an important area to focus on to improve FBT in the future, especially when families present with pre-existing communication, attachment problems or high level relationship difficulties [56]. Developing effective ways for the therapist to mediate distress and conflict may reduce criticism, a common barrier to successful engagement and outcome in FBT [10, 57].

The significant impact described on family relationships before treatment was not surprising and has been noted in other studies [5]. Adverse events, such as illness, have great potential to disrupt the attachment system goal for parents to be available and attuned to their adolescent’s emotional needs [58]. Therefore finding that closeness as well as improved communication occurred over the course of treatment is important finding given that effective parent-child relationships have important developmental consequences in terms of more positive psychosocial outcome [59]. The young people in this study reported improvements in their sense of self and confidence after treatment. These changes were directly connected to family changes during treatment for many, and indirectly by others as a product of the recovery process. Either way, this was a key finding as FBT’s longer-term goal is to reinstate a normal adolescent development trajectory as part of recovery [40].

The parents and adolescents who reported less relational change described particular premorbid family issues that they felt were difficult to change. Treatment in this study was manualized so augmenting FBT to address premorbid issues early in the process was not possible, as they were not preventing physical recovery. However, some of the families may have benefitted by addressing the relational barriers they were experiencing earlier in treatment. This is something the FBT manual does not currently address but it is becoming increasingly recognised that augmentation for particular patient groups is needed [60].

### Strengths

The strengths of this study include a severely unwell group of patients at the commencement of treatment. This patient group is generally difficult to engage in research and relatively little is known about their experiences. Treatment was manualized and fidelity confirmed by an author of the FBT manual ensuring more consistent observations than most other adolescent FBT qualitative studies to date. To our knowledge this is the first qualitative study that included both parents and patient from the same family allowing a more nuanced analysis of relationship changes to occur.

### Limitations

This study has a number of limitations including a relatively small sample size. While data reached saturation there is always the possibility that further interviews would have elicited new information. The purposive sampling strategy limits results to the experience of this specific group of participants, and it is likely that a favourable treatment experience contributed to their recruitment, and thus broader conclusions about the experience of all families undergoing FBT are not possible.

### Future research

Future research should continue to explore processes of change in FBT so that more is understood about those who partially respond or drop out and those who are treated in different treatment contexts such as specialist verses generalist outpatient care. Qualitative studies are well placed to identify issues with treatment experience that may lead to new treatment developments important augmentations to current recommendations. Particularly important where evidence is limited for alternatives to family therapy [21].

### Clinical implications

This study provides support for FBT’s capacity to protect or improve the relationship between adolescents and their parents over the course of treatment. These findings underscore the importance of concentrating on parental management of eating disorder symptoms as improved parental confidence in this area had an overlapping effect not just on physical health but improving security for the adolescent. Parental confidence was improved by fidelity to the FBT directives and therapists should recognise that these elements are not just practical strategies but provide parents with a secure base.

When relationship distress appears to be preventing progress, therapists may be able to improve the containment for adolescents by more directly discussing why parents are doing what they are doing. Likewise reframing adolescent anger and distress as care seeking may help parents take a less critical stance both during and away from meal times. Providing time in sessions to help adolescents’ articulate their experience, and supporting parents to listen in a validating way may be an important way to help the adolescent reengage and seek support, as long as the initial primary focus remains on physical recovery. A more direct focus on relationship issues in parallel to refeeding may also improve outcomes for some families.

## Abbreviations

CDC: Centre for disease control
DSMIV: Diagnostic and statistical manual of mental disorders
EBW: Estimated body weight
EDE: Eating disorder examination
FBT: Family based treatment.
